# Technical Note: validation of a material assignment method for a retrospective study of carbon-ion radiotherapy using Monte Carlo simulation

**DOI:** 10.1093/jrr/rrab028

**Published:** 2021-05-17

**Authors:** Weishan Chang, Yusuke Koba, Takuya Furuta, Shunsuke Yonai, Shintaro Hashimoto, Shinnosuke Matsumoto, Tatsuhiko Sato

**Affiliations:** Center for Radiation Protection Knowledge, National Institute of Radiological Sciences, QST, 4-9-1, Anagawa, Inage-ku, Chiba 263-8555, Japan; Center for Radiation Protection Knowledge, National Institute of Radiological Sciences, QST, 4-9-1, Anagawa, Inage-ku, Chiba 263-8555, Japan; Nuclear Science and Engineering Center, Japan Atomic Energy Agency, 2-4 Shirakata, Tokai-mura, Ibaraki 319-1195, Japan; Department of Accelerator and Medical Physics, National Institute of Radiological Sciences, QST, 4-9-1, Anagawa, Inage-ku, Chiba 263-8555, Japan; Nuclear Science and Engineering Center, Japan Atomic Energy Agency, 2-4 Shirakata, Tokai-mura, Ibaraki 319-1195, Japan; Department of Accelerator and Medical Physics, National Institute of Radiological Sciences, QST, 4-9-1, Anagawa, Inage-ku, Chiba 263-8555, Japan; Department of Accelerator and Medical Physics, National Institute of Radiological Sciences, QST, 4-9-1, Anagawa, Inage-ku, Chiba 263-8555, Japan

**Keywords:** carbon-ion radiotherapy (CIRT), material assignment method, Monte Carlo (MC) simulation, dose reconstruction

## Abstract

We propose a two-step method to converse human tissue materials from patient computed tomography (CT) images, which is required in dose reconstructions for a retrospective study of carbon-ion radiotherapy (CIRT) using Monte Carlo (MC) simulation. The first step was to assign the standard tissues of the International Commission on Radiological Protection reference phantoms according to the CT-number. The second step was to determine the mass density of each material based on the relationship between CT-number and stopping power ratio (Hounsfield unit [HU]-*SPR*) registered in treatment planning system (TPS). Direct implementation of the well-calibrated HU-*SPR* curve allows the reproduction of previous clinical treatments recorded in TPS without uncertainty due to a mismatch of the CT scanner or scanning conditions, whereas MC simulation with realistic human tissue materials can fulfill the out-of-field dose, which was missing in the record. To validate our proposed method, depth-dose distributions in the homogenous and heterogeneous phantoms irradiated by a 400 MeV/u carbon beam with an 8 cm spread-out Bragg peak (SOBP) were computed by the MC simulation in combination with the proposed methods and compared with those of TPS. Good agreement of the depth-dose distributions between the TPS and MC simulation (within a 1% discrepancy in range) was obtained for different materials. In contrast, fluence distributions of secondary particles revealed the necessity of MC simulation using realistic human tissue. The proposed material assignment method will be used for a retrospective study using previous clinical data of CIRT at the National Institute of Radiological Sciences (NIRS).

## INTRODUCTION

With the increasingly widespread use of carbon-ion radiotherapy (CIRT), there is a need to understand the long-term outcome of CIRT, specifically, the risk of secondary cancer [1–3]. On the other hand, these risk models have not yet been sufficiently verified by an epidemiological study with patient data because no such study using CIRT has been reported. The National Institute of Radiological Sciences of National Institutes for Quantum and Radiological Science and Technology (NIRS, QST) has possessed the follow-up data of more than 10 000 patients who received CIRT at NIRS with the general information recorded in clinical practice, which should be ideal for an epidemiological study.

Radiation-induced cancer risk increases with radiation doses, and the secondary cancer risk after radiotherapy can be estimated by the organ dose delivered by therapeutic radiation [4–6]. The pencil-beam algorithm has been implemented in the treatment planning systems (TPSs) of the NIRS as it has been for ion beam radiotherapies [7]. The pencil-beam algorithm can compute the dose distribution within a reasonable time for clinical application with a certain degree of accuracy for predicting the dose distribution in the planning target volume (PTV) and surrounding normal tissues. However, accuracy for predicting the dose distribution by pencil-beam algorithm is questionable for regions far from the PTV because it only partially considers the contribution of secondary particles. In fact, Kry *et al.* [8] reported that pencil-beam algorithm failed to predict the low-dose (< 5% of prescription dose) region. Therefore, even though dose distributions calculated by the TPS are recorded, it is insufficient for a retrospective analysis. To reconstruct the dose distribution, Monte Carlo (MC) simulation is the preferred method because it can trace all the particles produced during the transport. In fact, MC simulation has come to be used as a tool for independent dose calculation for patient specific quality assurance, or for benchmarking for dose calculation algorithms of commercial TPS [9–12].

**Fig. 1.**
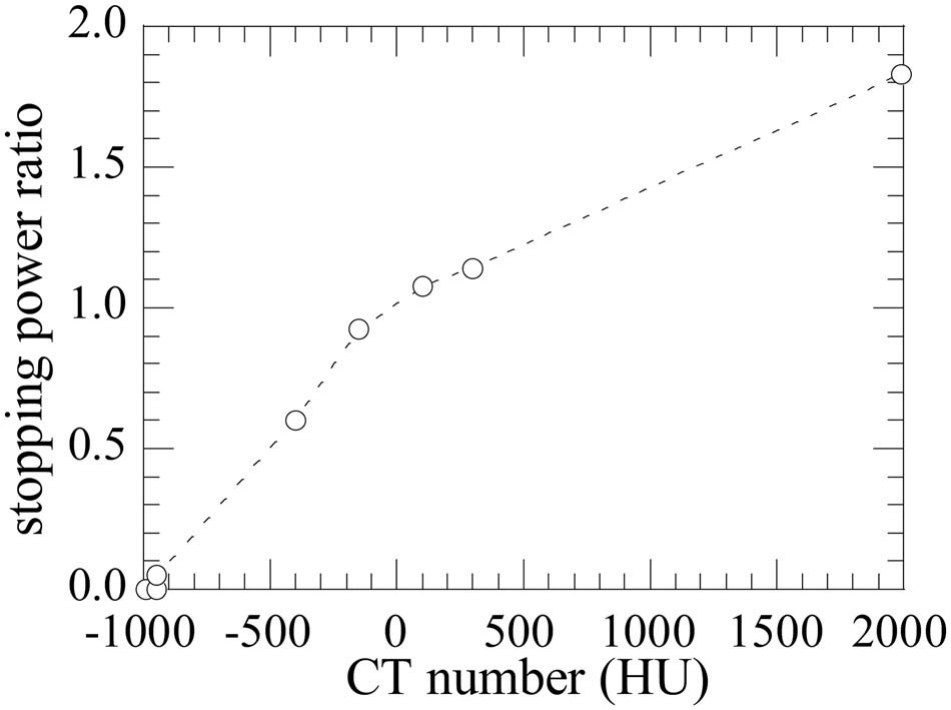
Example of HU-*SPR* curve implemented in TPS.

In pencil-beam algorithm, radiation fields are represented by integration of pencil beams, and dose profile of each pencil beam is computed by extending or compressing the pre-registered dose profile in water according to the map of the stopping power ratio (SPR) to water converted from the patient’s computed-tomography (CT) image [13]. The SPR is determined through a conversion curve between the CT-number (Hounsfield unit, HU) and SPR, known as the HU-*SPR* curve (Fig. 1), which is calibrated specifically for the CT machine by the scan of a set of known materials. On the other hand, MC simulation requires elemental composition and mass density of the materials to estimate the nuclear reactions and stopping power of traveling particles. A simple assumption is to take the elemental composition to be the same as water but vary the mass density to satisfy the SPR value. This method, called the ‘varying-density-water method’ hereafter, is occasionally adopted in a simplified dose assessment by MC simulation [14], but using water instead of real materials causes incorrect estimation of nuclear interactions. Up to now, most popular series of real-material based material assignment methods were based on an approach of Schneider *et al.* [15]. In this method, human tissue elemental compositions were adopted from Woodard and White [16] and assigned according to the CT-number. The mass density was also determined by the CT-number by using a fitting function based on CT scan data of several materials. These methods followed Schneider *et al*.’s methodology and derived a specific fitting function for each CT scanner and scanning condition [17–19]. Some of these methods have been implemented in TPSs [17,20–22].

However, the aim of the material assignment method for retrospective dose revaluation is rather different from these because the goal is to compensate the out-of-field region while reproducing the past treatment plan in PTV regions as much as possible. Specifically, minimization of discrepancies with the TPS in the stopping power of incident carbon ions is most important for the material assignment method for retrospective dose revaluation. We therefore propose a new method that is consistent with the HU-*SPR* curve in TPSs. Adoption of a well-calibrated HU-*SPR* curve may be crucial when recalibration is impossible because the CT system used for the CT image has been retired. A similar approach has been adopted in other studies [11,21]. However, our method is based on a direct integration of the HU-*SPR* curve of the TPS to be used and can be applied to any TPS of CIRT without additional correction factors.

The aim of this Technical Note was to describe our new material assignment method adopting the HU-*SPR* curve of the TPS and describe its validation performed by using homogeneous and heterogeneous phantoms. This method will be implemented in a new MC-based dosimetry system for CIRT, which will be used for assessment of previous clinical data of CIRT at the NIRS.

## MATERIALS AND METHODS

### Material assignment method

**Fig. 2.**
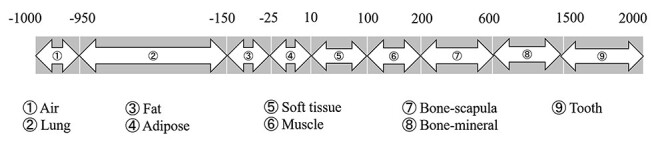
Allocation scheme of CT numbers and materials used in this work.

For transport calculation of MC simulation, information of the elemental composition and mass density of the materials is necessary. In this study, we adopted the elemental composition and elemental mass fraction of eight representative human tissue from ICRP Publication 110 [23] as shown in Table 1 and assigned these materials, including air, to the corresponding CT number interval with reference to Schneider *et al.* [15], as shown in Fig. 2.

**Table 1:** Elemental compositions and elemental mass fraction of the materials used in this work

Material	*w*_H_/%	*w*_C_/%	*w*_N_/%	*w*_O_/%	*w*_Na_/%	*w*_Mg_/%	*w*_P_/%	*w*_S_/%	*w*_Cl_/%	*w*_Ar_/%	*w*_K_/%	*w*_Ca_/%
**Air**	0.0	0.012	75.527	23.178	0.0	0.0	0.0	0.0	0.0	1.283	0.0	0.0
**Lung**	10.3	10.7	3.2	74.6	0.2	0.0	0.2	0.3	0.3	0.0	0.2	0.0
**Fat**	11.96	76.87	0.0	11.17	0.0	0.0	0.0	0.0	0.0	0.0	0.0	0.0
**Adipose**	11.4	58.8	0.8	28.7	0.1	0.0	0.0	0.1	0.1	0.0	0.0	0.0
**Soft tissue**	10.4	23.1	2.8	62.7	0.1	0.0	0.2	0.3	0.2	0.2	0.0	0.0
**Muscle**	10.2	14.2	3.4	71.1	0.1	0.0	0.2	0.3	0.1	0.0	0.4	0.0
**Bone-scapula**	8.7	30.9	2.6	48.3	0.2	0.1	3.0	0.4	0.2	0.0	0.0	5.6
**Bone-mineral**	3.6	15.9	4.2	44.8	0.3	0.2	9.4	0.3	0.0	0.0	0.0	21.3
**Tooth**	2.2	9.5	2.9	42.1	0.0	0.7	13.7	0.0	0.0	0.0	0.0	28.9

**Table 2:** Summary of the properties of calculation methods implemented in MC (our method and varying-density-water method) and TPS (pencil-beam algorithm)

		Material	Produced fragments
MC			
	our method	realistic tissues based on ICRP	realistic
	varying-density-water method	water with different *ρ*	fragments produced by interaction with water
TPS			
	pencil-beam algorithm	water with different *ρ*	No^*^

^*^The pencil-beam algorithm only considered the registered dose distribution in water that partially includes contributions of secondary particles.

The mass densities, which vary with HU, are determined by using the HU-*SPR* curve implemented in a TPS. The *SPR* of an arbitrary material to the water *SPR*_m, w_ for an incident carbon ion can be approximately expressed by using Bethe–Bloch theory, which can be expressed as:

(1) \begin{equation*} SP{R}_{\mathrm{m},\mathrm{w}}=\frac{{\left( dE/ dx\right)}_{\mathrm{m}}}{{\left( dE/ dx\right)}_{\mathrm{w}}}\cong \frac{\frac{\rho_{\mathrm{m}}}{u}\sum \limits_i\frac{w_i{Z}_i}{A_i}\left\{\ln \frac{m_e{c}^2}{I_{\mathrm{m}}}-\frac{1}{3}\right\}}{\rho_{\mathrm{w}}^e\left\{\ln \frac{m_e{c}^2}{I_{\mathrm{w}}}-\frac{1}{3}\right\}}, \end{equation*}

where the subscript m and w indicate the arbitrary material and water, respectively, ${\rho}_{\mathrm{w}}^e$ and ${\rho}_{\mathrm{m}}$ are the electron density of water and mass density of the material, respectively. *I* is the mean excitation energy, *u* is the unified atomic mass unit, m_e_*c*^2^ is the electron mass, *w_i_* is the elemental mass fraction for element *i* and *Z*_i_ and *A_i_* are the atomic number and relative atomic mass for element *i*, respectively. According to Bragg’s rule, *I*_m_ can be obtained by: $\ln{I}_m=\sum \limits_i\frac{w_i{Z}_i}{A_i}\ln{I}_i/\sum \limits_i\frac{w_i{Z}_i}{A_i}$,and *I*_w_ is adopted as 78 eV [24]. The factor 1/3 in equation (1) is adopted from Kanematsu *et al.* where the constant velocity of the incident carbon 0.557c is assumed because the change in *SPR*_m, w_ is < 0.5% for carbons with a residual range of 2 to 25 cm [25]. Equation (1) denotes that ${\rho}_{\mathrm{m}}$ can be uniquely determined from the CT number with the assignment of the elemental composition. Using equation (1), ${\rho}_{\mathrm{m}}$ for the corresponding CT number can be derived by:

(2) \begin{equation*} {\rho}_{\mathrm{m}}= SP{R}_{\mathrm{m},\mathrm{w}}\cdot u\cdot{\rho}_{\mathrm{w}}^e\left\{\ln \frac{m_e{c}^2}{I_{\mathrm{w}}}-\frac{1}{3}\right\}/\sum \limits_i\frac{w_i{Z}_i}{A_i}\left\{\ln \frac{m_e{c}^2}{I_{\mathrm{m}}}-\frac{1}{3}\right\}, \end{equation*}

where *SPR*_m, w_ is a function of the CT number as shown in Fig. 1 and thus ${\rho}_{\mathrm{m}}$ is determined from the HU-*SPR* curve provided by the TPS. It should be noticed that the HU-*SPR* curve is explicitly implemented in equation (2), so this method can be applied for any TPS of CIRT based on the HU-*SPR* curve without further complex correction processes required for other studies [11,21]. Only the *SPR* of incident carbon ions was considered in our method because doses delivered in the irradiation field mainly consist of carbon ions in CIRT [26], and the incident carbons primarily determine the distal edge of the dose distribution. In this work, we adopted the HU-*SPR* curve (Fig. 1) implemented in the TPS of the NIRS (XiO-N, ELEKTA, Stockholm, Kingdom of Sweden and Mitsubishi Electric, Tokyo, Japan).

We decided to vary ${\rho}_{\mathrm{m}}$ every 10 HU between −1000 HU and 2000 HU although ${\rho}_{\mathrm{m}}$ can vary continuously with increasing CT number in principle according to equation (2). This is because implementing the ${\rho}_{\mathrm{m}}$ increments corresponds to each CT number requiring an extremely large amount of memory and has high computational costs. Barnes *et al.* investigated the relationship between density bin size and the range tolerance for lung, soft tissue and bone. The range error for a proton beam was found to be 0.13 mm and 1.01 mm for densities of 1.00 g cm^−3^ (soft tissue) and 0.26 g cm^−3^ (lung), respectively, when the CT noise was 9 HU and 493 density bins were adopted [27]. Considering the range error and resolution of the CT image, a 10 HU step was sufficient for our study. In this case, the range error for a mono-energy carbon beam was evaluated within 0.2 mm for water.

**Table 3:** Properties for the eight materials used in the validation. *SPR*_m,w_ is obtained from the HU-*SPR* curve implemented in TPS by interpolation, ${\rho}_{\mathrm{m}}$ and ${\rho}_{\mathrm{w}}$ are mass densities derived using equation (2) for the method and varying-density-water method, respectively

	Lung	Fat	Adipose	Soft tissue	Muscle	Bone-Scapula	Bone-Mineral	Tooth
**HU**	−325	−105	−5	45	175	265	715	1585
***SPR***_**m,w**_	0.70	0.95	1.01	1.04	1.10	1.13	1.31	1.66
***ρ***_**m**_	0.70	0.92	0.98	1.04	1.10	1.16	1.47	1.92
***ρ***_**w**_	0.70	0.95	1.01	1.04	1.10	1.13	1.31	1.66

It should be mentioned that special care is necessary for CT images containing streak/metal artifacts. The dosimetric impact from these artifacts for heavy charged particle radiotherapy is higher than that from photon radiotherapy because of the larger influence on the range due to the variation of the Bragg peak positions, in addition to dose difference on the artifacts. Joost [28] has studied dosimetric impact of metal artifacts in proton radiotherapy and found that the dose/range errors mainly occurred around the distal end of the Bragg peak. On the other hand, Jackel [29] concluded that beam paths intersecting the streak artifacts lead to errors in the range of around or below 1% which seem to be less critical for proton/ion radiotherapy. It is suggested to review the CT image and the treatment plan before retrospective analysis because the electron density or relative stopping power of soft tissue is assigned manually to the region affected by artifacts in some cases. Therefore, the inclusion of such information is our future work.

### Validation of the proposed methods using the Monte Carlo simulation

To validate our proposed material assignment method, transport calculations of a 400 MeV/u carbon beam with an 8 cm spread-out Bragg peak (SOBP) in homogeneous and heterogeneous phantoms were performed using a MC simulation code, and depth-dose distribution inside the phantoms were computed. In addition, a transport calculation by replacing the materials with water but varying mass density to match the stopping power of incident carbons (equation 2) was also performed to demonstrate deficiencies of varying-density-water method applying to detailed analysis of secondary particles. The results were compared with those of the TPS (XiO-N, ELEKTA). Table 2 summarizes the characteristics of our proposed method, varying-density-water method and pencil-beam algorithm.

The MC simulation code used in this work was the Particle and Heavy-ion Transport Code System (PHITS) ver. 3.02 [30] which has been used in simulations for CIRT in several studies [31–34], and accuracies of this code for various research fields, including medical physics, have been verified [35]. For the physical models and libraries used in this work, we followed the recommendation for particle therapy in the PHITS package. The transport of photons and electrons are calculated by EGS5 [36] and nuclear reactions between light ions are calculated using the INCL model [37]. Nuclear collisions of a projectile are described by the quantum molecular dynamics model JQMD [38] and the reaction cross-section model Kurotama [39]. The stopping power of the charged particles is calculated by the ATIMA code (http://web-docs.gsi.de/∼weick/atima/). The energy threshold for production/transport of secondary particles in the MC simulations was set as 1 MeV for all charged particles.

MC simulation computed the dose deposited in the material through the stopping power and mass density of the material. The computed dose for a material defined by realistic-tissue is called the dose to matter *D*_m_. On the other hand, the computed dose for a material defined by varying-density-water is called the dose to water *D*_w_. *D*_w_ slightly deviates from *D*_m_ because of the difference in elemental compositions between actual human tissue and water. Since the dose obtained by the TPS is *D*_w_, to compare dose distributions between the TPS and MC simulation with realistic-tissue based material, conversion from *D*_m_ to *D*_w_ is necessary. We derived *D*_w_ as the summation of energy deposited by each imparted particle *i* per unit of mass, and *D*_w_ is therefore expressed by the following equation:

(3) \begin{equation*} {D}_{\mathrm{w}}=\frac{\sum \limits_i{\left({\varepsilon}_{\mathrm{w}}\right)}_i}{m}=\frac{\sum \limits_i{\left({\varepsilon}_{\mathrm{w}}\right)}_i}{V{\rho}_{\mathrm{w}}}=\frac{1}{V{\rho}_{\mathrm{m}}}\sum \limits_i\frac{1/{\rho}_{\mathrm{w}}}{1/{\rho}_{\mathrm{m}}}{\left[\frac{\varepsilon_{\mathrm{m}}\cdot{\left(\mathrm{d}E/\mathrm{d}x\right)}_{\mathrm{w}}}{{\left(\mathrm{d}E/\mathrm{d}x\right)}_{\mathrm{m}}}\right]}_i \end{equation*}

where *ε*_w_ is the energy deposited in water, *ε*_m_ is the energy deposited in the medium, *V* is the tally volume, *m* is its mass and *ρ*_w_ and *ρ*_m_ are the mass densities of the water and medium, respectively. It should be noted that (d*E*/d*x*)_w_ and (d*E*/d*x*)_m_ change depending on the particle species and particle energies and should be specifically computed for each particle *i* in this method.

For the homogeneous phantom, a 30 × 30 × 40 cm^3^ rectangular box composed of the eight representative human tissue materials listed in Table 1 was used. A representative CT number was chosen from the interval shown in Fig. 2 for each material and listed in Table 3. The corresponding SPR, *SPR*_m,w_, mass density ${\rho}_{\mathrm{m}}$ and water density ${\rho}_{\mathrm{w}}$ are also listed in Table 3. The phantom was irradiated by the carbon beam with a sufficiently large field size covering the phantom, and the depth-dose distribution along the central beam axis was recorded by a cylindrical scoring mesh with a 2 cm radius and 0.1 mm step size. Difference in the peak positions (position of the maximum dose in the depth-dose distribution) between the MC simulation and TPS Δ_peak_ = peak_MC_—peak_TPS_ and the 50% dose positions (position where the depth-dose becomes 50% of the maximum dose) Δ_d50_ = d50_MC_—d50_TPS_ were evaluated to quantify the difference in the distributions.

**Fig. 3.**
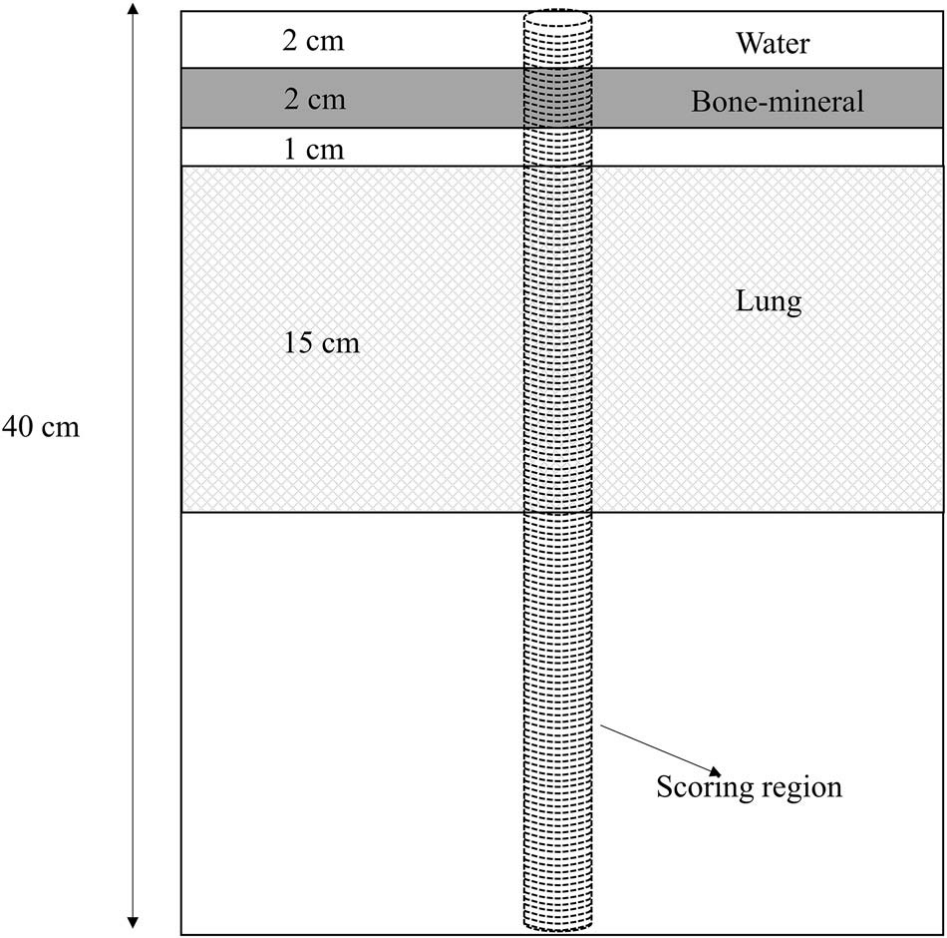
Schematic view of the 30 × 30 × 40 cm^3^ (external dimension) heterogeneous layer phantom.

For the heterogeneous phantom, a 30 × 30 × 40 cm^3^ rectangular geometry composed of layers of different materials in which a 2 cm bone-mineral (HU = 715) slab and a 15 cm lung (HU = −325) slab were inserted into a water phantom (H_2_O with mass density of 1.00 g cm^−3^), as schematically depicted in Fig. 3, was prepared. Elemental composition and mass density of the bone-mineral and lung are shown in Table 3. This geometry is prepared to mimic the situations of beam transport in a patient body which is mixture of materials with high and low CT numbers and to elucidate differences between MC simulations with realistic materials and varying-density-water method. The properties of the bone-mineral slab and lung slab were taken to be same as defined in Table 3. The scoring region was the same as that used for the homogeneous phantom. To quantify the difference in the depth-dose distributions, a gamma index (γ) analysis [40] was performed in addition to the evaluation of Δ_peak_ and Δ_d50_. The American Association of Physicists in Medicine’s report TG-218 recommends evaluation criteria based on a distance-to-agreement (DTA) of 2 mm and a dose difference of 3%. On the other hand, we imposed a more severe criteria; a DTA of 1 mm and a dose difference of 3% since we used a simple geometry.

**Fig. 4.**
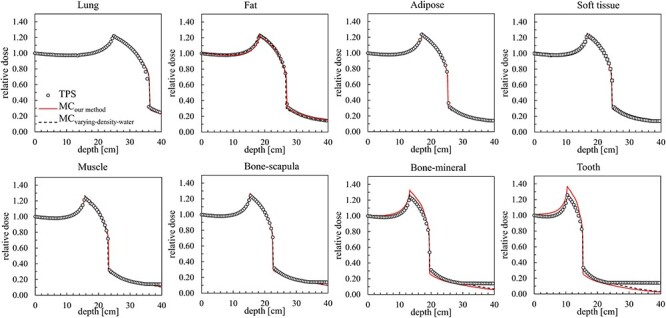
Comparison of dose distributions in eight different materials. Black circle, red solid line and black dash line represent the results from the TPS, MC simulation with implementation of our method (MC_our method_) and varying-density-water method (MC_varying-density-water_), respectively.

### Probability of nuclear reaction for the material assignment methods

For the dosimetry of CIRT, nuclear reactions are also of importance in addition to stopping power properties in matter because they affects the attenuation of primary carbon ions and the production of secondary particles. Nuclear reactions differ by elemental compositions. Thus nuclear reactions in varying-density-water are different from those in human tissue, although the SPRs were equalized by adjusting the water density ${\rho}_{\mathrm{w}}$. The non-linear relationship between the *SPR*_m,w_ and the ratio of the probability of nuclear interactions in a material to water ${\rho}_{\mathrm{N}}$ for carbon ions indicates that attenuation rates of the incident carbon beam cannot be scaled by the *SPR*_m,w_ [41,42]. Consequently, doses deposited by the incident carbon beam became different. The ${\rho}_{\mathrm{N}}$ has been formulated as [41].

(4) \begin{equation*} {\rho}_{\mathrm{N}}={\rho}_m\sum \limits_i{w}_i\frac{\sigma_{Ni}}{A_{\mathrm{r}i}}/{\left(\frac{\sigma_N}{A_{\mathrm{r}}}\right)}_{\mathrm{w}}, \end{equation*}

(5) \begin{equation*} {\left(\frac{\sigma_{\mathrm{N}}}{A_{\mathrm{r}}}\right)}_{\mathrm{w}}=\frac{2{\sigma}_{\mathrm{N}\mathrm{H}}+{\sigma}_{\mathrm{N}\mathrm{O}}}{M_{\mathrm{r}\mathrm{w}}}, \end{equation*}

where ${\sigma}_{\mathrm{N}}$ represents the nucleus–nucleus collision cross-section between a projectile nucleus of mass number *A* and a target nucleus of *A*_r*i*_ of element *i*, ${\sigma}_{\mathrm{NH}}$ and ${\sigma}_{\mathrm{NO}}$ are the nucleus–nucleus collision cross-sections of hydrogen and oxygen, respectively; and *M*_rw_ = 18.015 is the molecular weight of water. Sihver [43] formulated ${\sigma}_{\mathrm{N}}$ as:

(6) \begin{equation*} {\sigma}_{\mathrm{N}}=\pi{r}_0^2{\left[{A}^{1/3}+{A}_{\mathrm{r}i}^{1/3}-{b}_{0i}\left({A}^{-1/3}+{A}_{\mathrm{r}i}^{-1/3}\right)\right]}^2, \end{equation*}

where *r*_0_ = 1.36 fm is the effective nucleon radius and *b*_0i_ is the overlap parameter given by:

(7) \begin{equation*} {b}_{0i}=\left\{\begin{array}{@{}l}2.247-0.915\left(1+{A}_{\mathrm{r}i}^{-1/3}\right)\kern2.24em \mathrm{for}\kern0.33em \mathrm{protons}\\{}1.581-0.876\left({A}^{-1/3}+{A}_{\mathrm{r}i}^{-1/3}\right)\kern1em \mathrm{for}\kern0.33em \mathrm{ions},\end{array}\right. \end{equation*}

From equations (2) and (4–7), it is apparent that *SPR*_m,w_ is proportional to ${\rho}_{\mathrm{N}}$ for varying-density-water method, whereas it is not linear in reality and with our method. In other words, the probabilities of nuclear interactions of two different materials can be different even the *SPR*s are the same.

To evaluate the influence of material assignment method on nuclear reaction, we computed the fluence of primary particle (^12^C) and secondary particles (proton, helium and neutron) in the scoring region shown in Fig. 3.

## RESULTS

### Depth-dose distribution in the homogeneous phantom

Figure 4 shows a comparison of depth-dose distributions in the homogeneous phantom made of eight different materials. All of the depth-dose distribution curves were normalized at the entrance of the phantom. Black circle, red solid line and black dashed line represent results of the TPS, MC simulation with implementation of our method (MC_our method_) and varying-density-water method (MC_varying-density-water_), respectively. Table 4 summarizes dose differences in the peak Δ_peak_ and in the 50% dose positions Δ_d50_ between the TPS, MC_our method_ and MC_varying-density-water_.

### Depth-dose distribution in the heterogeneous phantom

**Fig. 5.**
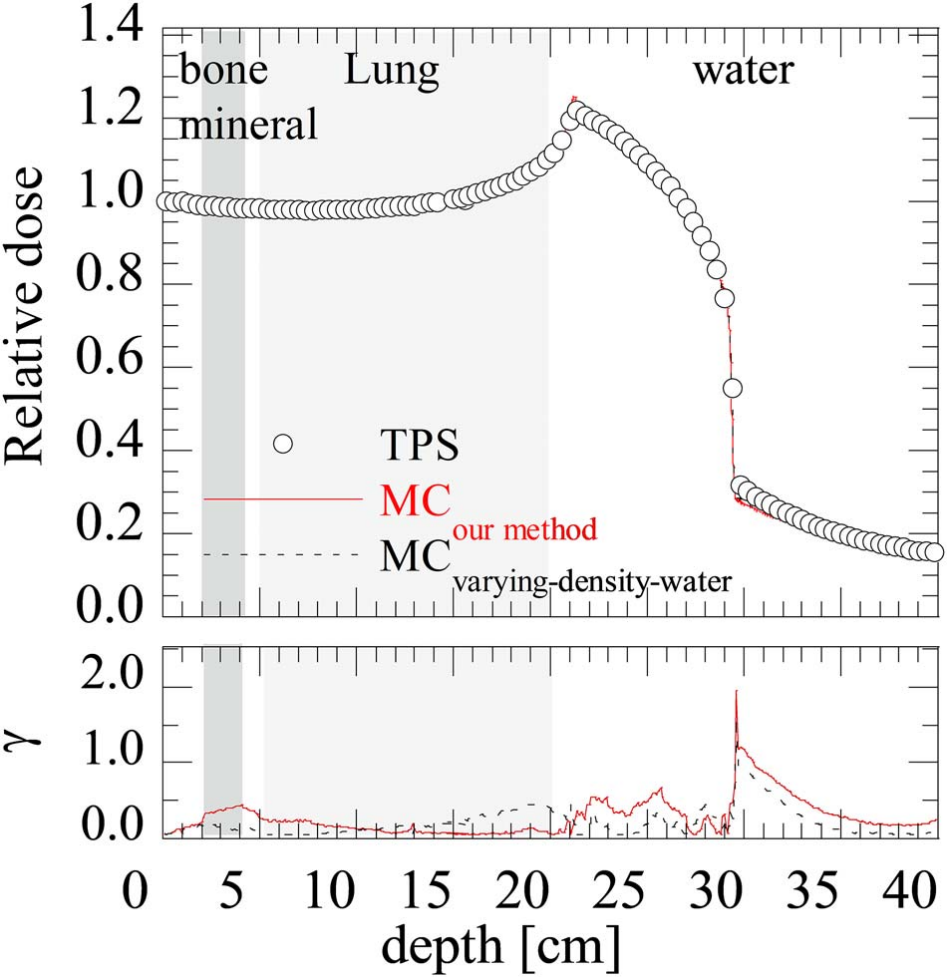
Comparison of depth-dose distributions in a heterogeneous layer phantom and gamma index between MC simulation and TPS. Black circle indicates the result from the TPS. Red solid line and black dashed line indicate the results of MC_our method_ and MC_varying-density-water_, respectively. The gamma passing rates for our method and the varying-density-water method for the 1 mm/3% criteria were 97.5% and 99.8%.

Figure 5 shows a comparison of depth-dose distributions in the heterogeneous phantom calculated by the TPS, MC_our method_ and MC_varying-density-water_. The distributions were normalized to 1.0 at the entrance of the phantom. The results of the gamma index analysis for the depth-dose distributions of the MC simulation were also given in Fig. 5 by taking the distribution of the TPS in the base. Table 5 shows the Δ_peak_ and Δ_d50_ between the TPS, MC_our method_ and MC_varying-density-water_.

**Table 4:** Differences to the TPS for the peak and the 50% dose points

		Lung	Fat	Adipose	Soft tissue	Muscle	Bone-scapula	Bone-mineral	Tooth
Our method	Δ_peak_ [mm]	–2.0	1.1	0.4	–0.3	–0.1	–0.9	–0.4	–0.3
	Δ_d50_ [mm]	2.8	1.0	–1.3	–1.4	0.9	–0.9	–0.8	–0.7
Varying-density-water method	Δ_peak_ [mm]	–1.6	–0.6	0.9	–0.1	–1.2	–0.9	–0.4	–0.2
	Δ_d50_ [mm]	2.9	–1.4	–1.1	–0.9	–1.3	–0.8	–0.6	–0.4

**Table 5:** Differences to the TPS in the case of the heterogeneous phantom

	Δ_peak_ [mm]	Δ_d50_ [mm]
Our method	0.6	–0.5
Varying-density-water method	0.3	–0.3

### Probability of nuclear reaction for the material assignment methods

**Fig. 6.**
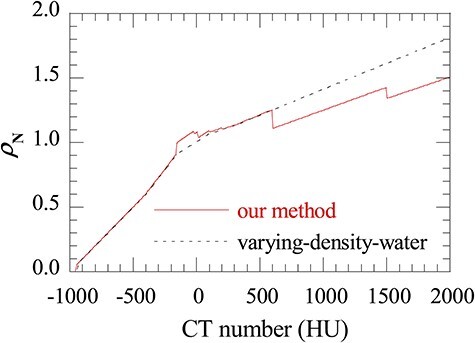
The relationship between CT number and ${\rho}_{\mathrm{N}}$ of carbon ions for materials defined by our method and the varying-density-water method.

Figure 6 shows the relationship between the CT number and ${\rho}_{\mathrm{N}}$, where ${\rho}_{\mathrm{N}}$ is calculated according to the equations (4–7) and the materials are defined by varying-density-water method and our method, respectively. ${\rho}_{\mathrm{N}}$ by varying-density-water method was smaller than that by our method when the CT number is between −150 HU and 10 HU, which corresponds to the material ‘fat’ and ‘adipose,’ whereas an opposite tendency that ${\rho}_{\mathrm{N}}$ by varying-density-water method was larger than that by our method when CT number is larger than 600 HU, and the difference increases with CT number.

**Fig. 7.**
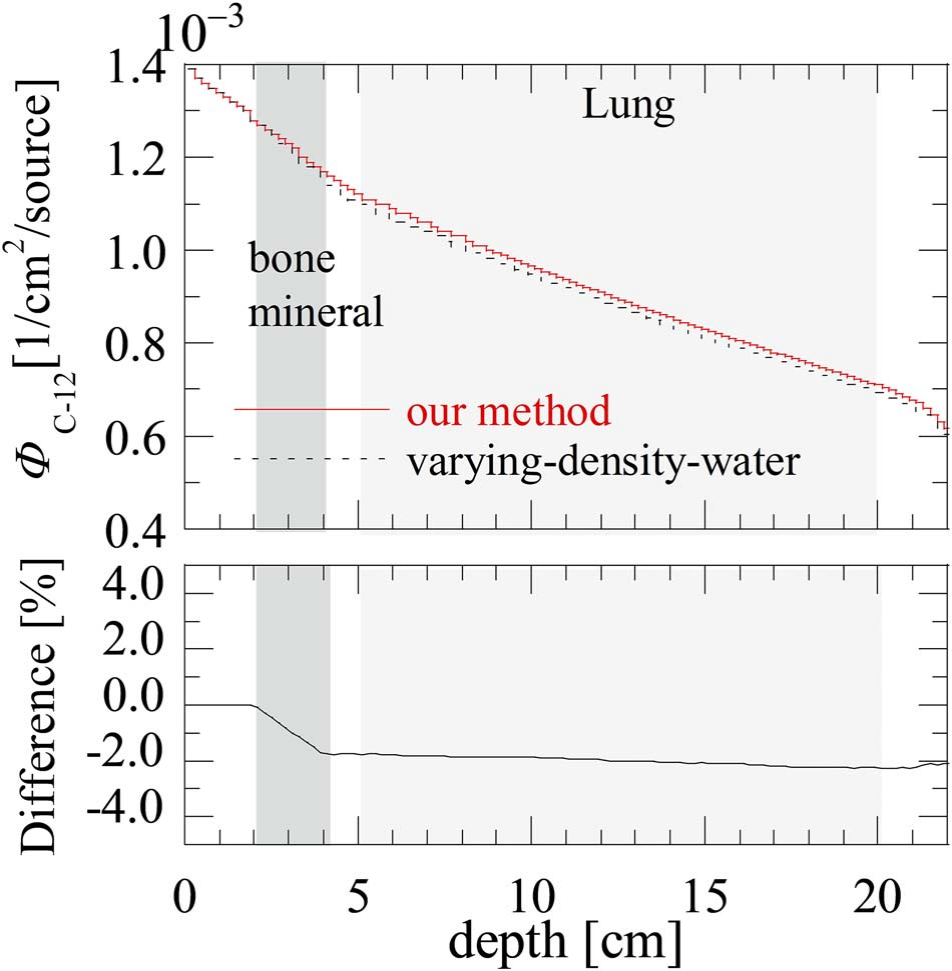
${\varPhi}_{\mathrm{C}-12}$ and the difference of ${\varPhi}_{\mathrm{C}-12}$ between MC_our method_ and MC_varying-density-water_ in the heterogeneous phantom.

**Fig. 8.**
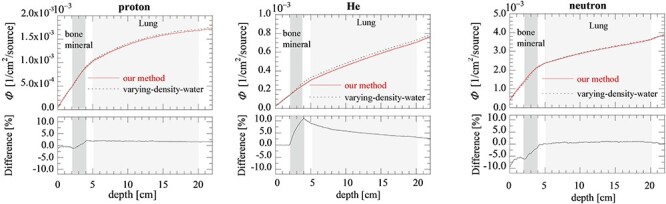
The secondary particle fluence (${\varPhi}_{\mathrm{proton}},\kern0.33em {\varPhi}_{\mathrm{He}}\kern0.33em \mathrm{and}\kern0.33em {\varPhi}_{\mathrm{neutron}}$) produced in the heterogeneous phantom when implementing our method and varying-density-water method.

### Particle fluence in the heterogeneous phantom

Figure 7 shows the fluence of ^12^C ${\varPhi}_{\mathrm{C}-12}$ and the difference of ${\varPhi}_{\mathrm{C}-12}$: (${\varPhi}_{\mathrm{C}-12}$ by varying-density-water − ${\varPhi}_{\mathrm{C}-12}$ by our method)/${\varPhi}_{\mathrm{C}-12}$ by our method. ${\varPhi}_{\mathrm{C}-12}$ by varying-density-water method becomes smaller than that by our method after passing through the bone-mineral region.

Figure 8 shows the secondary particle fluences (${\varPhi}_{\mathrm{proton}},{\varPhi}_{\mathrm{He}}\kern0.33em \mathrm{and}\kern0.33em {\varPhi}_{\mathrm{neutron}}$) in the heterogeneous phantom when implementing our method and varying-density-water method. The lower figures indicates the difference of those fluences defined similar to Fig. 7. The variations in the fluence in the bone-mineral region were largest for these three particles.

## DISCUSSION

Elongation of the depth-dose distribution varies with different materials. The peak position shifted shallower for materials with higher CT numbers because the SPR monotonically increases with increasing CT number as seen in the HU-*SPR* curve (Fig. 1). Depth-dose distributions in the homogeneous phantoms obtained by MC_varying-density-water_ almost perfectly reproduce those of the TPS as expected because of their conceptual similarity except for the far edge of the fragment tail regions (Fig. 4). This discrepancy was due to the limitation of the registered dose profile in the TPS. The TPS used in this study (XiO-N, ELEKTA) only possesses the depth-dose profile with a ≤ 40-cm depth in water-equivalent length and the dose level is kept constant beyond this depth. The depth-dose distributions obtained by MC_our method_ also match with those of the TPS and MC_varying-density-water_ for materials with lower CT numbers, although there were some discrepancies; there were higher doses around the peak positions and lower doses at the fragment tail regions, for materials with higher CT numbers (bone-mineral and tooth). These discrepancies are due to differences in nuclear reactions between high-density water and human tissue. Further discussion will be provided in the next paragraph. Despite these discrepancies, the positions of the peak and 50% dose of the MC_our method_ agreed well with those of the TPS and MC_varying-density-water_ for most of the materials except for the lung material (Table 4). The deviation for the lung material was mainly due to adaption of a 10-HU step to convert the mass density explained in Sec. 2.1 and was insignificant in comparison with the traveling distance of approximately 30 cm. We therefore confirmed that distal-edge position of the dose distribution by TPS can be reproduced by our method. The depth-dose distribution in the heterogeneous phantom obtained by MC_our method_ reproduces those of the TPS and MC_varying-density-water_ within 1 mm in the range (Fig. 5 and Table 5). The gamma-index analysis with DTA of 1 mm and a dose difference of 3% (Fig. 5 below) shows that the gamma passing rates for MC_our method_ and MC_varying-density-water_ were 97.5% and 99.8%, respectively. These rates are better than the usual clinical acceptance level of 95% [44], and the validity of our new material assignment method was confirmed by this example.

Large discrepancy in the nuclear reaction probability in high CT materials observed in Fig. 6 is consistent with the conclusion suggested by Matsufuji [45] that using material with a similar composition of bone is necessary for simulating the physical parameters, such as multiple scattering and nuclear interaction. This result explains the discrepancies between MC_our method_ and MC_varying-density-water_ seen in the depth-dose distributions (Fig. 4) for materials with higher CT numbers (bone-mineral and tooth).

According to the particle fluence analysis, we found that difference in ${\varPhi}_{\mathrm{C}-12}$ between MC_our method_ and MC_varying-density-water_ increased to 2% in the bone-mineral region and remained almost constant (difference ≦ 0.5%) in the lung region. The change of tendency can be understood by the difference of ${\rho}_{\mathrm{N}}$, where varying-density-water method underestimates ${\rho}_{\mathrm{N}}$ for high-CT materials such as bone-mineral while it predicts almost equivalent ${\rho}_{\mathrm{N}}$ for low-CT materials such as lung. Underestimation of ${\rho}_{\mathrm{N}}$ for high-CT materials in varying-density-water method leads to lower secondary particle productions rates in total and attributes the underestimates of doses at the fragment tail regions seen in Fig. 4.

Difference in elemental compositions also affects the proportions of secondary particles. Overestimates of ${\varPhi}_{\mathrm{He}}$ and underestimates of ${\varPhi}_{\mathrm{neutron}}$ for the bone-mineral in varying-density-water method can be understood as follows. Lighter fragments such as He as well as protons are mostly produced in the nuclear reactions between carbon ions and water elements composed only by protons and oxygens while more various fragments are produced in realistic bone-mineral material containing larger mass elements, which are also more likely to produce neutrons compared to the water elements. Discrepancy of neutron fluence in the water region in front of bone mineral is due to the backscattered neutrons produced in the bone mineral. The difference in secondary-particle fluence can potentially change the beam quality, such as averaged LET, which should be important for the retrospective analysis study. Therefore we concluded that varying-density-water method is not compatible with the detailed analysis for secondary particles and the necessity of MC simulations with realistic human tissue.

## CONCLUSION

With the aim of developing a revaluation tool for the treatment plan in CIRT using MC simulation, we proposed a new method to converse a realistic-tissue material from the CT number that is dedicated to reconstructing a patient body from the CT image. The relationship between the CT number and SPR in the TPS was accounted for by implementing the HU-*SPR* curve of the TPS directly in the key function. Adoption of a well-calibrated HU-*SPR* curve in the TPS allows reproduction of the TPS dose distribution around the PTV by avoiding discrepancies due to mismatch of the CT scanner or scanning conditions. In addition, the secondary particle dose can be properly estimated by adopting realistic-tissue materials instead of water. The validity of our method was tested by MC simulation in homogenous and heterogeneous phantoms. We confirmed that our method is suitable for implementing in the MC dosimetry system for retrospectively analyzing previous clinical data of CIRT at the NIRS.

## CONFLICT OF INTEREST

The authors declare they have no conflict of interest.
